# Macacine Herpesvirus 1 in Long-Tailed Macaques, Malaysia, 2009–2011

**DOI:** 10.3201/eid2107.140162

**Published:** 2015-07

**Authors:** Mei-Ho Lee, Melinda K. Rostal, Tom Hughes, Frankie Sitam, Chee-Yen Lee, Jeffrine Japning, Mallory E. Harden, Anthony Griffiths, Misliah Basir, Nathan D. Wolfe, Jonathan H. Epstein, Peter Daszak

**Affiliations:** EcoHealth Alliance, New York, New York, USA (M-.H. Lee, M.K. Rostal, T. Hughes, C.-Y. Lee, J.H. Epstein, P. Daszak);; Department of Wildlife and National Parks Peninsular Malaysia, Kuala Lumpur, Malaysia (F. Sitam, J. Japning, M. Basir);; Texas Biomedical Research Institute, San Antonio, Texas, USA (M.E. Harden, A. Griffiths);; Metabiota, San Francisco, California, USA (N.D. Wolfe)

**Keywords:** Macacine herpesvirus 1, long-tailed macaque, Macaca fascicularis, herpes B virus, encephalitis, zoonoses, PCR, occupational risk, translocation, nonhuman primate, viruses, Malaysia, B virus

## Abstract

Macacine herpesvirus 1 (MaHV1; B virus) naturally infects macaques (*Macaca* spp.) and can cause fatal encephalitis in humans. In Peninsular Malaysia, wild macaques are abundant, and translocation is used to mitigate human–macaque conflict. Most adult macaques are infected with MaHV1, although the risk for transmission to persons who handle them during capture and translocation is unknown. We investigated MaHV1 shedding among 392 long-tailed macaques (*M. fascicularis*) after capture and translocation by the Department of Wildlife and National Parks in Peninsular Malaysia, during 2009–2011. For detection of MaHV1 DNA, PCR was performed on urogenital and oropharyngeal swab samples. Overall, 39% of macaques were shedding MaHV1 DNA; rates of DNA detection did not differ between sample types. This study demonstrates that MaHV1 was shed by a substantial proportion of macaques after capture and transport and suggests that persons handling macaques under these circumstances might be at risk for exposure to MaHV1.

## Abstract 2

Virus shedding by 39% of wild-caught macaques creates potential occupational risk for humans.

Macacine herpesvirus 1 (MaHV1; also known as B virus) is a zoonotic pathogen that is enzootic among macaque (*Macaca* spp.) populations throughout Asia (*1*,*2*). MaHV1 is an α-herpesvirus related to human herpes simplex viruses (HSV) 1 and 2 (*3*,*4*) and to herpesviruses that infect other nonhuman primates such as baboons (*5*). Like HSV infection in humans, MaHV1 infection in macaques can clinically appear as vesicular lesions on the mucous membranes of the buccal cavity and genital area (*6*,*7*). However, macaques without clinically apparent lesions can still shed MaHV1 (*6*). 

Transmission of MaHV1 can occur transcutaneously (via bites) or permucosally (via exposure to macaque body fluids) (*8*,*9*). Among humans, ≈40 cases of MaHV1 encephalitis have been reported; all patients were laboratory workers who had come in contact with rhesus macaques (*M. mulatta*) only or with rhesus macaques and long-tailed macaques (*M. fascicularis*) or their tissues in the research environment (*2*,*3*). For these patients, signs and symptoms of MaHV1 infection included skin ulcers and lesions at the site of injury, influenza-like illness, and infection of the peripheral and central nervous systems (which can develop into brainstem encephalomyelitis and death) (*7*,*9*). The mortality rate for humans with untreated MaHV1 infection is >70% (*7*). This high case-fatality rate has led to strict regulations for handling macaques and macaque clinical samples in laboratories and resulted in the designation of MaHV1 as a Biosafety Level 4 (BSL-4) pathogen and, until recently, a select agent (*2*,*7*,*9*).

In macaques, MaHV1 frequently remains latent in the trigeminal and lumbosacral ganglia; however, in response to stress, it can be asymptomatically reactivated and shed in saliva and urogenital excretions (*10*). Macaques typically acquire MaHV1 at sexual maturity (*11*); previous studies have found IgG against MaHV1 in up to 100% of sexually mature wild or laboratory long-tailed and rhesus macaques (*11*,*12*). As with other viral infections, the presence of IgG indicates previous exposure or infection but does not indicate active virus shedding. During active infection, MaHV1 DNA can be detected in saliva or urogenital samples by use of PCR. Virus culture is also possible but is not routinely performed because doing so safely requires a BSL-4 laboratory (*11*). Using PCR as a diagnostic method has advantages over culture in that it can be performed under BSL-2 conditions, it produces results more rapidly, and its sensitivity and specificity are higher (*13*,*14*).

Little is known about the shedding rate of MaHV1 in macaques outside the laboratory setting, the frequency of transmission to humans, or the incidence of MaHV1 encephalitis among humans (particularly those with frequent contact with macaques). In Asia, at least 50% of cases of encephalitis are never diagnosed to the point of causative agent identification (*15*). Understanding the ecology of MaHV1 among macaques is essential for understanding the potential for human infection. Macaques have adapted to urbanized human environments, and contact between humans and macaques can occur in a variety of contexts (e.g., feeding in public recreational areas, capture of wild macaques for the pet trade or biomedical research colonies, consumption, or population management by wildlife authorities). Human–macaque contact can result in bites, scratches, and indirect exposure to macaque body fluids (*16*,*17*). Simian foamy virus, a nonpathogenic retrovirus found in nonhuman primates including macaques, has been transmitted during occupational exposure to macaques via bites and scratches in many of the aforementioned contexts and in agricultural, suburban, and urban environments (*18*–*20*). Zoonotic transmission of simian foamy virus to humans has been demonstrated in Indonesia (*18*) and Bangladesh (*19*). Because exposure to MaHV1 can occur through similar routes (*8*), its transmission under circumstances similar to those of transmission of simian foamy virus in Asia is plausible.

In Peninsular Malaysia, conflict between humans and macaques in residential and public areas results from loss of macaque habitat, successful macaque adaptation to human environments, and subsequent macaque overpopulation. As a result, the Department of Wildlife and National Parks (DWNP) in Peninsular Malaysia implemented a macaque population management program, which includes the removal or translocation of macaques from a conflict area. The possibility of exposure to MaHV1 during macaque capture and transport presents a potential occupational hazard to wildlife personnel.

Our aim with this study was to describe the prevalence of MaHV1 shedding among wild-caught long-tailed macaques after capture and transport in Peninsular Malaysia. This study represents a step toward understanding the potential for zoonotic transmission of MaHV1 outside the laboratory.

## Materials and Methods

### Capture and Sample Collection

Independently of this study, DWNP, as part of their macaque management program throughout Peninsular Malaysia, captured and transported macaques from 6 states (Johor, Perak, Pahang, Pulau Pinang, Selangor, and Negeri Sembilan) to DWNP holding facilities. Capture and opportunistic blood sampling was performed by DWNP and EcoHealth Alliance during September–November 2009, July–October 2010, and July 2011. The macaques captured had been free ranging and lived in the peripheral vegetation of rural, suburban, and urban communities in several states of Peninsular Malaysia. Trapped animals were transferred into transport cages and taken to the nearest local DWNP facility, where they were held up to 72 h before being transported to the DWNP headquarters in Kuala Lumpur or relocated to a new area. Macaques were kept in groups in cages and provided with food and water throughout the holding period. Animals were captured in accordance with the protocols and guidelines of the Manual for Human–Macaque Conflict Management in Peninsular Malaysia (*21*). This study was conducted under Institutional Animal Care and Use Committee approval no. 18048 from the University of California (Davis, CA, USA). When handling and sampling macaques, personnel involved with this study wore personal protective equipment (PPE; e.g., eye protection, double-layered nitrile gloves, Tyvek coveralls, and P100 respirators) (*22*). Blood and swab samples were collected from each animal at its arrival at the headquarters or at the local DWNP facility before relocation. Macaques were immobilized with an intramuscular injection of a combination of 5 mg/kg ketamine and 5 mg/kg xylazine (*21*). After immobilization, oropharyngeal swab and urogenital swab samples were collected (when possible, urine was also collected by cystocentesis). The samples were placed in 2 mL cryovials (Nalgene Nunc International, Rochester, NY, USA) with 500 μL NucliSens lysis buffer (bioMérieux, Marcy l’Étoile, France) and immediately stored at −80°C. Macaque weight, body condition, sex, and approximate age were recorded. The age of the animals was determined by assessing their weight, body size, and the development of their incisors and genitals (*23*). Macaques were categorized as adult (>7 years), subadult (3–6 years), or juvenile (1–3 years). The sex and age of 2 animals and the sex of 1 adult animal were not recorded.

### Molecular Testing 

The samples were vigorously mixed; 100 μL of the sample was used for mechanical nucleic acid extraction by use of the NucliSENS miniMAG system (bioMérieux). The extracted nucleic acid was eluted with 60 μL of buffer. PCR was performed as previously described and validated by Scinicariello et al. (*13*). Briefly, MaHV1 primers (B virus 1, 5′-ACCTCACGTACGACTCCGACT-3′; and B virus 2, 5′-CTGCAGGACCGAGTAGAGGAT-3′; 2.5 μmol/L) were each added to the extraction product and HotStarTaq Plus Master Mix (QIAGEN, Hilden, Germany). The product was placed in a thermocyler at 94°C for 5 min and then underwent 30 cycles as follows: 94°C for 1 min, 56°C for 1 min, and 72°C for 1 min. The products (10 μL) were then analyzed by electrophoresis on 1% agarose gels. Of the ≈10% of samples that were positive by PCR, 14 PCR products were randomly selected and purified with a PCR purification kit (QIAGEN) and sequenced by using the same primers to confirm identity. Sequences were 128 bp and were analyzed by using a BLAST search of GenBank (*24*). Because PCR is reported to be highly specific (*13*) and all 14 PCR products showed 93%–100% nucleotide homology to MaHV1, we considered the other PCR products with identical amplicon size to also be positive for MaHV1. PCR is more sensitive than culture for detecting HSV (*25*,*26*), and we considered the detection of MaHV1 DNA in a sample as an indication of virus shedding, although viral load was not obtained through culture or quantitative PCR.

The positive control was produced in a BSL-4 facility and removed from containment by use of inactivation procedures approved by the Texas Biomedical Research Institute Biohazard Committee. Briefly, macacine herpesvirus 1 strain E2490 virion “mini-prep” DNA was generated as previously described (*27*). With the same B virus 1 and 2 primers, the region between nt 54886 and 54993 was amplified by PCR from the viral genome (*13*) by using the FailSafe PCR Enzyme Mix (Epicenter, Madison, WI, USA). This region corresponded to a region in the *UL28* open reading frame. The resulting 128-nt fragment was cloned into pCR2.1-TOPO by using the TOPO TA kit (Invitrogen, Carlsbad, CA, USA) to generate the *pMHUL28* gene. The insert was sequenced and confirmed.

PCR sensitivity (limit of detection) was determined by using DNA from *pMHUL28* and 2 confirmed-positive samples by diluting the DNA to copy numbers of 2.71 × 10^4^ for *pMHUL28* and 1.59 × 10^11^ for the samples. The sensitivity limit for *pMHUL28* by PCR was 9.13 × 10^2^ molecules, and for the swab samples it was ≈1 × 10^4^ molecules. The PCR sensitivity was previously determined to be ≈100 gene copies by using purified viral DNA, and specificity was determined by *SacII* restriction enzyme analysis and Southern blot hybridization by using an MaHV1-specific internal probe (5′-GGAGAAGACGTCGCGGTCGTAC-3′) that discriminates MaHV1 from HSV (*13*).

### Immunoassay

A subset of 149 animals, randomly chosen to represent each age group, were tested by the MaHV1 ELISA as described by Ohsawa et al. (*28*). Although the exact specificity and sensitivity of the original MaHV1 ELISA was not determined, it had been validated by testing of known MaHV1-positive (n = 14) and negative (n = 6) serum, and the assay correctly detected 100% of the positive samples and provided negative results for 100% of the negative samples (R. Eberle, pers. comm., 2013). In brief, MaHV1-infected and noninfected cell antigens were added to 96-well round-bottom plates and prepared as previously described (*28*). Wells were blocked with phosphate-buffered saline containing 5% bovine serum albumin and 0.05% Tween 20 (PBS-BSA-Tw) and were incubated at 37°C for 1 h, then rinsed with PBS-Tw. Serum samples were diluted to 1:100 with PBS-BSA-Tw, added to the plate (50 μL/well), and incubated at room temperature for 2 h. The wells were washed 5 times with PBS-Tw. Biotinylated anti-human IgG (Vector Laboratories, Burlingame, CA, USA) was diluted 1:5,000 with PBS-BSA-Tw (50 μL/well) and incubated at room temperature for 1 h. The wells were washed 5 times with PBS-Tw. A complex of avidin and biotinylated peroxidase was prepared according to the manufacturer’s instructions, diluted to 1:32, added to washed wells (50 μL/well), and incubated at room temperature for 1 h. The wells were washed 5 times with PBS-Tw. A 3,3′,5,5′-tetramethylbenzidine substrate solution was added (100 μL/well), and the plates were incubated without light for 8–12 min. The reaction was stopped with 2 mol/L sulfuric acid (50 μL/well), and the optical density at 450 nm was measured by using a microplate absorbance reader (Bio-Rad Laboratories, Hercules, CA, USA). An ELISA result was considered positive when the optical density was >0.1 (*28*).

### Statistical Analyses

We calculated standard prevalence rates and 95% CIs (*29*) for differences in shedding prevalence based on macaque sample type, sex, and age. For pairwise analysis (2 parameters) of proportions, we conducted a *z*-test. A general linear model was used to investigate the effect of geographic location by state, sex, and age, and the Akaike Information Criterion was used to select the best-fit model (with no significant interactions between the variables). An analysis of variance of the general linear model with a post hoc Tukey HSD (honest significant difference) test was used to assess the significance of each factor. Statistical analyses were conducted by using the R Statistical package (R Core Team, Vienna, Austria). A p value of <0.05 was considered statistically significant.

## Results

Samples from 392 long-tailed macaques from 6 states within Peninsular Malaysia (Figure) were screened by PCR; 149 of these were also screened by ELISA (Table 1). The overall detection of MaHV1 DNA in macaques, in urogenital and/or oropharyngeal samples (n = 392 tested), was 39.3% (95% CI 34.5%–44.1%). All 14 sequenced DNA samples displayed 93%–100% homology with those of MaHV1. Shedding status did not differ significantly among age groups: 37.6% (95% CI 31.2%–43.9%) among 221 adults, 38.8% (95% CI 28.1%–49.4%) among 80 subadults, and 43.8% (95% CI 33.5%–54.1%) among 89 juveniles (for 2 animals, age was not recorded). Male macaques were more likely than females to be shedding the virus at the time of sampling; prevalence was 44.1% (95% CI 37.5%–50.7%) among 220 males and 33.1% (95% CI 26.0%–40.2%) among 169 females; the sex of 3 animals was not recorded (*z*-statistic = 2.192466, degrees of freedom [df] = 1, p = 0.0001) (Table 1). Males were also significantly more likely than females to shed virus in saliva; prevalence was 26.4% (95% CI 20.5%–32.2%) among 220 males and 16.0% (95% CI 10.5%–21.5%) among 169 females (*z*-statistic = 2.457458, df = 1, p = 0.007). Overall, the proportion of urogenital and oropharyngeal samples positive for MaHV1 DNA did not differ significantly: 24.7% (95% CI 20.5%–29.0%) of urogenital samples and 21.9% (95% CI 17.8%–26.0%) of oropharyngeal samples were positive. We detected viral DNA in oropharyngeal and in urogenital swabs for 18.8% (95% CI 12.7%–25.0%) of the 154 macaques with positive results by PCR (Table 2).

**Figure:**
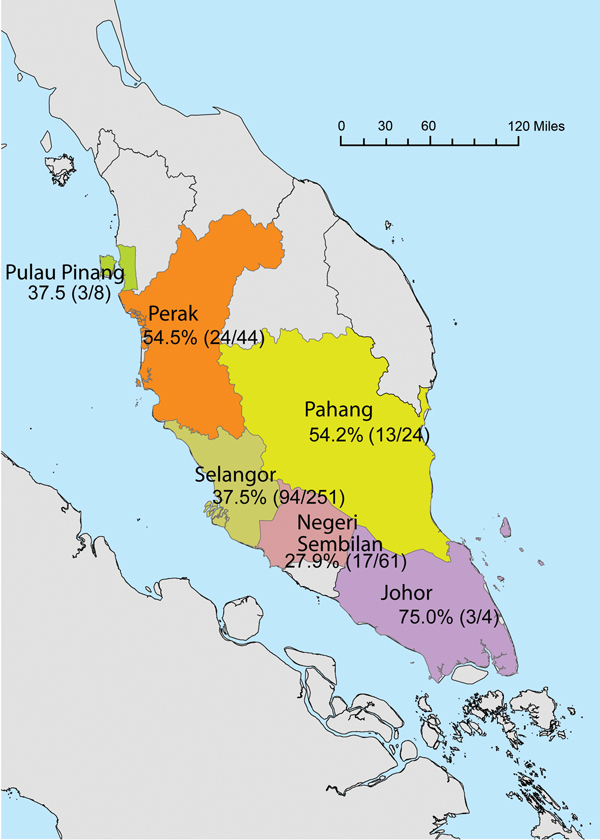
State of origin and prevalence of macacine herpesvirus 1 shedding within sampled groups of macaques (no. positive/total tested) from Peninsular Malaysia, September 2009–July 2011.

**Table 1:** PCR results for macacine herpesvirus 1 in macaques, by age and sex. Malaysia, 2009–2011*

Age group, y	Male			Female			Unspecified†			Total	
	No.	No. (%; 95% CI) positive		No.	No. (%; 95% CI) positive		No.	No. (%) positive		No.	No. (%; 95% CI) positive
Adult, >6	120	55 (45.8; 36.9–54.7)		100	28 (28.0; 19.2–36.8)		1	0		221	83 (37.6; 31.2–43.9)
Subadult, 3–6	48	19 (39.6;25.7–53.4)		32	12 (37.5; 20.7–54.3)		0	0		80	31 (38.8; 28.1–49.4)
Juvenile, <3	52	23 (44.2; 30.7–57.7)		37	16 (43.2; 27.3–59.2)		0	0		89	39 (43.8; 33.5–54.1)
Unspecified†	0	0		0	0		2	1 (50.0)		2	1 (50.0; NA)
Total	220	97 (44.1; 37.5–50.7)‡		169	56 (33.1; 26.0–40.2)‡		3	1 (33.3)		392	154 (39.3; 34.5–44.1)

*NA, not applicable.  †2 macaques of unspecified age and sex and 1 adult macaque of unspecified sex were included in the study. ‡Indicates a significant difference (p<0.05) between the 2 groups marked.

**Table 2:** PCR results for macacine herpesvirus 1 in macaques, by sample type, Malaysia, 2009–2011*

Sex	Oropharyngeal			Urogenital			Oropharyngeal and urogenital	
	No.	No. (%; 95% CI) positive		No.	No. (%; 95% CI) positive		No.	No. (%; 95% CI) positive
M	220	58 (26.4; 20.5–32.2)†		220	56 (25.5; 19.7–31.2)		97	17 (17.5; 10.0–25.1)
F	169	27 (16.0; 10.5–21.5)†		169	41 (24.3; 17.8–30.7)		56	12 (21.4; 10.7–32.2)
Unknown‡	3	1 (33.3; 0–86.7)		3	0		0	0
Total	392	86 (21.9; 17.8–26.0)		392	97 (24.7; 20.5–29.0)		29 (18.8)	29 (18.8; 12.7–25.0)

*For animals with positive results for both oropharyngeal and urogenital samples, percentages are of the total number of positive animals. †Indicates a significant difference (p<0.05) between the 2 groups marked for oropharyngeal swabs. ‡3 macaques of unspecified sex were included in the results.

Overall, IgG against MaHV1 was found in 73 (49.0%; 95% CI 38.5%–57.0%) of 149 macaques. We found that seroprevalence differed significantly among age groups: IgG was found in 70.0% of 50 adults, 46.0% of 50 subadults, and 30.6% of 49 juveniles (χ^2^ = 15.6333, df = 2, p = 0.0004) (Table 3). Among macaques tested by ELISA, 24.2% (95% CI 17.3%–31.0%) were positive according to PCR but negative according to ELISA results, although these animals did not differ significantly by age, sex, or shedding site (oropharyngeal vs. urogenital) (Table 4).

**Table 3:** ELISA results for macacine herpesvirus 1 antibodies in , by age and sex, Malaysia, 2009–2011

Age group, y	Male			Female			Unspecified			Total	
	No.	No. (%; 95% CI) positive		No.	No. (%; 95% CI) positive		No.	No. (%; 95% CI) positive		No.	No. (%; 95% CI) positive
Adult, >6*	32	21(65.6; 49.2–82.1)		17	13 (76.5; 60.0–96.6)		1	1 (100.0; NA)		50	35 (70.0; 53.5–82.7)
Subadult, 3–6*	29	12 (41.4; 23.5–59.3)		21	11 (52.4; 34.5–73.7)		0	0 (NA)		50	23 (46.0; 28.1–59.8)
Juvenile, < 3*	26	8 (30.8; 13.0–48.5)		23	7 (30.4; 12.7–49.2)		0	0 (NA)		49	15 (30.6; 12.9–43.5)
Total	87	41 (47.1; 36.6–57.6)		61	31 (50.8; 40.3–63.4)		1	1 (100.0; NA)		149	73 (49.0; 38.5–57.0)

*Indicates a significant difference (p<0.05) between the age groups marked.

**Table 4:** ELISA and PCR results for macacine herpesvirus 1 in macaques, by age, Malaysia, 2009–2011

Age group, y	No. animals	No. (%) animals			
		PCR positive, ELISA positive	PCR positive, ELISA negative	PCR negative, ELISA positive	PCR negative, ELISA negative
Adult, >6	50	10 (20.0)	8 (16.0)	25 (50.0)	7 (14.0)
Subadult, 3–6	50	9 (18.0)	13 (26.0)	14 (28.0)	14 (28.0)
Juvenile, <3	49	4 (8.2)	15 (30.6)	11 (22.4)	19 (38.8)
Total	149	23 (15.4)	36 (24.2)	50 (33.6)	40 (26.8)

The geographic origin of macaques that were MaHV1 positive by PCR was as follows: 4 (75.0%; 95% CI 32.6%–100%) were from Johor, 44 (54.5%; 95% CI 39.8%–69.3%) from Perak, 24 (54.2%; 95% CI 34.2%–74.1%) from Pahang, 8 (37.5%; 95% CI 4.0%–71.0%) from Pulau Pinang, 251 (37.5%; 95% CI 31.5%–43.4%) from Selangor, and 61 (27.9%; 95% CI 16.6%–39.1%) from Negeri Sembilan (Figure). An analysis of variance of the general linear model indicated that both sex and geographic location (state) were significantly associated with detection by PCR, but only the effect of sex (F-statistic = 3.97, df = 1, p = 0.047) had enough power to remain significant after a post hoc Tukey HSD (for categorical data) was applied (p = 0.049).

## Discussion

We examined MaHV1 shedding among free-ranging macaques after capture and transport, a scenario under which occupational exposure could occur. In addition to the risk that capture and transport poses for handlers, macaques are probably under increased physiologic stress during capture and transport, which might result in increased virus activation and shedding.

Despite little published data for shedding prevalence in free-ranging or recently captured wild macaques with which to compare our findings, serologic evidence from wild-caught macaques transported from India to the United States in the 1950s for polio vaccine testing indicates that the stress of transport probably led to increased MaHV1 seroprevalence from 10% before transit to 70% after transit (*30*). These animals were young (1.0–1.5 years of age) and were kept in groups of 60. PCR studies of laboratory macaques (rhesus and long-tail) have reported a shedding prevalence range of 0%–71% (*11*,*14*,*31*). Sample sizes in these studies were generally very small, and most reported prevalence rates were <10%. Shedding prevalence determined in our study certainly falls within these ranges.

We observed that shedding prevalence was significantly higher among male than female macaques, although seroprevalence did not differ. This difference could be related to sociobiological dominance behavior by which females typically remain at the dominance level of their mother, whereas males lose that dominance rank when they leave the group at the time of dispersal. Thus, males continually must earn their ranking as they change groups (*32*). This behavior might predispose males to greater social stress during capture and transport. Unfortunately, we were unable to separate sex-based differences in physiologic stress from the potential effects of the stress of capture and transport, which might have affected male macaques differently than females.

We observed a significant difference in seroprevalence among macaques in different age groups; seroprevalence was highest among adults. This finding was consistent with findings of previous studies (*11*,*12*); however, we did not observe an age-based difference in shedding prevalence. One potential bias in our sampling strategy was that the age groups, which were composed of randomly selected individuals, did not reflect the age ratio of the overall group, which might have contributed to the lack of difference among age groups in shedding prevalence. We had expected to see a lower rate of shedding among juvenile than among adult animals because seroconversion is evident at sexual maturity for most laboratory and free-ranging macaques (*33*). It could be that younger animals were experiencing primary infection from exposure during capture and transport, which could explain the higher than expected shedding prevalence for this age group and in the overall study.

Our primary aim with this study was detection of MaHV1 DNA in macaques. However, we included serologic test results from a subset of animals to identify antibody seroprevalence among macaques in different age groups and to determine whether shedding occurs in the absence of detectable antibodies. We detected viral DNA in 36 seronegative macaques. This finding may have been the result of a recent primary infection before detectable IgG response. Some animals could have been infected by conspecifics during transport or just before capture. It is also possible that these animals were experiencing acute virus reactivation resulting from the stress of capture and transportation during the 6–72 h before sampling because previously infected animals would probably be seropositive. Unfortunately, data for the duration of time between capture and sampling were not available. The incubation period for HSV-1 or HSV-2 in humans is 2–12 days (*34*). Although the time between onset of stress and MaHV1 reactivation has not been determined for macaques, in mice, HSV can reactivate in as little as 14 h after exposure to a stressor (*35*). Approximately 80% of the animals shedding MaHV1 in the absence of detectable IgG were subadults or juveniles, suggesting that this infection was their first.

The low frequency of simultaneous MaHV1 detection in oropharyngeal and urogenital swab samples suggests a variable shedding pattern among individuals, which was not unexpected given the fact that the virus can sequester itself in the ganglia of the trigeminal nerve, sacral nerve (*36*,*37*), or both, which would probably affect the route of virus excretion. Other MaHV1 studies have also reported inconsistent detection of virus in oral and genital secretions from infected laboratory macaques sampled repeatedly over time (*11*,*31*). Sensitivity of the PCR assay we used was lower than that of the one used by Scinicariello et al. (*13*), which might have resulted in underdetection of viral DNA in macaque clinical samples. Use of real-time PCR (not available for this study), such as that developed by Huff et al. (*38*), which has a sensitivity of 10 viral particles, would substantially improve sensitivity of future studies that screen macaques for MaHV1.

The observed MaHV1 shedding patterns suggest that a substantial proportion of animals shed virus after, and potentially during, transport and that the risks for exposure to MaHV1 by wildlife personnel or others handling macaques under these circumstances should be seriously considered. Appropriate PPE, including coveralls, gloves, N95 or P100 respirators, and eye protection, are recommended for wildlife personnel when handling macaques (and any other nonhuman primate) under conditions in which stress and prolonged confinement with other macaques may contribute to increased shedding of MaHV1 and potentially other pathogens. Indeed, as a result of this study, DWNP is strengthening its existing policies requiring personnel handling macaques to wear PPE and use proper work area biosafety and disinfection techniques to reduce the risk for transmission of MaHV1 and other zoonotic pathogens, in accordance with established safety protocols (*22*). Personnel working with macaques have received additional training to increase their awareness of the potential risks for exposure to MaHV1. 

Future studies should determine whether zoonotic transmission has occurred among those who have occupational contact with macaques during procedures such as capture, sample collection, treatment, and translocation (e.g., wildlife personnel) and should determine the incidence rate for infection among high-risk populations. Questions remain about the etiology of viral encephalitides throughout Asia and what proportion of these may be caused by MaHV1. Studies that examine the shedding prevalence of MaHV1 in free-ranging macaques will improve our understanding of shedding in the absence of anthropogenic stressors and, coupled with human surveillance, will enable further assessment of the potential risk for zoonotic transmission. These results will be of particular relevance to professionals who are occupationally exposed to macaques. 
